# Variation and Interdependencies of Human Milk Macronutrients, Fatty Acids, Adiponectin, Insulin, and IGF-II in the European PreventCD Cohort

**DOI:** 10.3390/nu11092034

**Published:** 2019-08-30

**Authors:** Maria Grunewald, Christian Hellmuth, Franca F. Kirchberg, Maria Luisa Mearin, Renata Auricchio, Gemma Castillejo, Ilma R. Korponay-Szabo, Isabel Polanco, Maria Roca, Sabine L. Vriezinga, Katharina Werkstetter, Berthold Koletzko, Hans Demmelmair

**Affiliations:** 1 Ludwig-Maximilians-Universität, Division of Metabolic and Nutritional Medicine, Dr. von Hauner Children’s Hospital, University of Munich Medical Center, 80337 Munich, Germany; 2 Department of Paediatrics, Leiden University Medical Center, 2300 Leiden, The Netherlands; 3 Department of Medical Translational Sciences and European Laboratory for the Investigation of Food-Induced Diseases, University Federico II, 80131 Naples, Italy; 4 Department of Pediatric Gastroenterology Unit, Hospital Universitari Sant Joan de Reus, URV, IIPV, 43201 Reus, Spain; 5 Celiac Disease Center, Heim Pál Children’s Hospital, 1089 Budapest, Hungary; 6 Department of Pediatric Gastroenterology and Nutrition, La Paz University Hospital, 28033 Madrid, Spain; 7 U. Enfermedad Celiaca e Inmunopatología Digestiva, Instituto de Investigación Sanitaria La Fe, 46026 Valencia, Spain

**Keywords:** human milk, celiac disease, hormones, fatty acids, duration of lactation, country, carbohydrate, fat

## Abstract

Human milk composition is variable. The identification of influencing factors and interdependencies of components may help to understand the physiology of lactation. In this study, we analyzed linear trends in human milk composition over time, the variation across different European countries and the influence of maternal celiac disease. Within a multicenter European study exploring potential prevention of celiac disease in a high-risk population (PreventCD), 569 human milk samples were donated by women from five European countries between 16 and 163 days postpartum. Some 202 mothers provided two samples at different time points. Protein, carbohydrates, fat and fatty acids, insulin, adiponectin, and insulin-like growth factor II (IGF-II) were analyzed. Milk protein and n-6 long chain polyunsaturated fatty acids decreased during the first three months of lactation. Fatty acid composition was significantly influenced by the country of residence. IGF-II and adiponectin concentrations correlated with protein content (*r* = 0.24 and *r* = 0.35), and IGF-II also correlated with fat content (*r* = 0.36), suggesting a possible regulatory role of IGF in milk macronutrient synthesis. Regarding the impact of celiac disease, only the level in palmitic acid was influenced by this disease, suggesting that breastfeeding by celiac disease mothers should not be discouraged.

## 1. Introduction

Breastfeeding supports physiological infant growth and development [1]. The importance of early life nutrition has been stimulated in studies investigating human milk composition and influencing factors [2,3,4,5]. A recent meta-analysis found that the average energy content in human milk of mothers with term born babies hardly changes from lactation week 2 to weeks 10–12 [6]. However, at both time points, the energy content shows large inter-individual variation. This primarily reflects a high variation of milk fat content, but also protein and to a lesser extent lactose are variable [7]. Colostrum and transitional milk are clearly different from mature milk. After the second week of lactation, changes associated with the duration of lactation, like the decrease in protein content, only partially explain the variation in milk composition and other influencing factors, for example, maternal diet, have to be considered [7]. 

The fatty acid (FA) composition of human milk fat is dependent on maternal diet. This has been demonstrated for essential FA and their long chain polyunsaturated derivatives (LC-PUFA) [8], as well as for medium chain FA (MCFA, C8.0 to C14.0) contents in milk, which are influenced by the ratio of dietary carbohydrates to fat [9]. Milk protein is composed of casein and whey, which is mainly comprised by α-lactalbumin and lactoferrin, but also includes a variety of lower concentrated proteins and peptides [10]. Insulin, insulin-like growth factors, and adipokines are metabolic regulators that might modulate infantile metabolism after milk feeding [11,12]. The hormones in milk may be derived from the maternal circulation, as suggested for insulin [13], or they could be synthesized in the mammary gland [11]; and their concentrations may be related to other human milk components. Co-variation of peptide hormone and macronutrient concentrations in human milk might complicate the identification of growth promoting or growth attenuating effects to individual compounds. This could also in part explain why studies observing the relationship between human milk composition and infant growth often yield ambiguous results [14,15,16,17]. 

Celiac disease (CD) is an intolerance of gluten, a protein present in various cereals. The disease is associated with atrophy of the intestinal villi, inflammation of the jejunal mucosa, and intestinal malabsorption [18]. A lifelong gluten free diet (GFD) is required to improve the histopathology and symptoms of CD, such as steatorrhea, diarrhea, and abdominal distension [18]. However, there is a risk that adherence to a GFD induces nutritional deficiencies, as GFDs have been found to be low in iron, calcium, B-vitamins, and some fatty acids [19]. There are ambiguous findings in relation to the effect of a GFD on fatty acid status biomarkers [20,21]. It is currently not known whether human milk fatty acid composition is affected by maternal CD. So far, it has only been shown that CD affects cytokines in milk [22]. Significant effects of CD or GFD on macronutrient contents or fatty acid composition could be of importance for the nutrition of breast fed infants of CD mothers and might require specific dietary recommendations. 

In this study, we determined protein, fat, carbohydrate, individual FA, insulin, adiponectin, and insulin-like growth factor II (IGF-II) in milk samples collected in the large European PreventCD prospective cohort study. We aimed to compare milk composition between mothers with CD and healthy mothers, to investigate any effects by country of residence and duration of lactation on milk composition and to analyze the variation and interdependencies of the measured milk components. 

## 2. Materials and Methods

Human milk samples were collected from 2007 to 2010 within the PreventCD study [23]. Details on the study population are reported in Vriezinga et al. [24]. Briefly, healthy newborns were enrolled if they had at least one first-degree family member with biopsy-confirmed celiac disease and were tested positive for the risk alleles *HLA-DQ2* and/or *HLA-DQ8*. Infants born preterm or with any congenital disorder were excluded. Infants were randomized to the introduction of either small amounts of gluten or to placebo at the age of 16 weeks. 

The PreventCD study was approved by the medical ethics committee of each participating center and complied with Good Clinical Practice guidelines (ICH-GCP) regulations. The study was conducted according to the Declaration of Helsinki. The PreventCD Current Controlled Trials number is ICTRP CTRP NTR890.

Milk samples for this study were donated by mothers in five European countries between 16 days and 163 days postpartum. The included milk samples were collected in the Netherlands (Leiden, *n* = 116), Italy (Naples, *n* = 68), Spain (Madrid, Valencia, and Barcelona, *n* = 138), Hungary (Budapest, *n* = 120), and Germany (Munich, *n* = 127). 

Mothers were asked to express milk manually or by pump once a month during the first six months after birth without further specification for fore- or hind-milk sampling and time of day. Milk samples were first frozen at −20 °C in home freezers, transferred to the hospital on ice, and then stored at −80 °C. Samples for the reported analyses were aliquoted (1–2 mL) and randomly selected, aiming for two samples from each mother, with one sample collected until 3 months postpartum (early samples), and one sample collected during months 4 or 5 (late samples). 

### 2.1. Measurements

Analytical procedures were previously described in a publication observing the association between milk components and the infant metabolome [25]. Measurement of total fat and total carbohydrates was performed via mid-infrared spectroscopy with a Human Milk Analyzer (MIRIS AB, Uppsala, Sweden) [26]. Owing to limited available sample volumes, the samples were diluted 1:3 with water. Samples were sonicated and heated to 40 °C prior to analysis. Tests with a diluted reference milk sample revealed intra- and inter-assay coefficients of variation (CVs) (7 and 13 determinations) for fat (5.3% and 6.6%) and carbohydrates (4.8% and 4.5%), comparable to the inter-assay CVs of undiluted milk samples (fat: 5.6% and carbohydrates: 4.3%). The calibration curve of eight different diluted samples versus the same eight undiluted samples showed high correlations with R² of 0.99 for fat and 0.90 for carbohydrates, respectively.

As the protein measurement by infrared spectroscopy (MIRIS) led to unsatisfactory CVs, the protein content was measured with an adapted Bradford method [27]. The intra batch—and inter batch—assay CVs of 4 and 16 determinations were calculated with 4.3% and 9.7%, respectively, using samples with 1.3 g/dL protein. Spiking recovery was determined to be 99.1% ± 27.6% in eight low (+0.27 g/dL) and 105.8% ± 16.5% in eight high (+0.44 g/dL) spiked samples.

Analysis of the FA composition of milk lipids was performed as previously described using 20 µL of milk [28]. The lipid bound FAs were converted in situ with acidic catalysis into FA methyl esters, which were subsequently extracted into hexane and analyzed by gas chromatography. The method enabled quantification of FA with 8 to 24 carbon atoms, including the major LC-PUFA. The weight percentages of 35 FA were determined with a mean CV of 4.9%, as estimated from 31 analyses of control milk aliquots measured along with study samples.

For the analysis of hormones, milk aliquots were thawed overnight at 4 °C and skimmed by centrifugation at 4000× *g* and 4 °C for 30 min. Total adiponectin concentration was measured with a commercially available ELISA kit (Biovendor RD191023100 High Sensitivity Adiponectin, Brno, Czech Republic) in 50 µL skimmed milk with a 1:3 dilution following the protocol of the manufacturer. The intra-batch and inter-batch CVs of 4 and 8 determinations were 4.5% and 4.8%, respectively. Spiking recovery was found to be 105.1% ± 14.0% in eight low (+2 ng/mL) and 91.6% ± 4.0% in eight high (+10 ng/mL) spiked determinations.

Insulin was measured with the Mercodia Insulin ELISA kit 10-1113-01 (Mercodia, Uppsala, Sweden) from 25 µL of undiluted, skimmed human milk, according to the protocol of the manufacturer. The intra-batch and inter-batch CVs of 4 and 8 determinations were 3.4% and 11.0%, respectively. Spiking recovery was determined to be 92.3% ± 14.8% in seven low (+21 mU/L) and 85.9% ± 7.2% in seven high (+42 mU/L) spiked samples.

IGF-II was determined with a radioimmunoassay from 30 µL of full fat milk by Mediagnost (Reutlingen, Germany) using the R-30 IGF-II RIA kit, according to the protocol of the manufacturer. The kit had already successfully been applied for the analysis of IGF-II in human milk [29]. 

### 2.2. Data Analysis

In order to evaluate the effects of duration of lactation and country of residence, data were divided into subsets of early (day 16–100) and late (day 101–163) lactation. Statistical analyses were performed independently on both subsets, that is, separately on the early and late samples. We identified outliers by calculating the numeric distance to its nearest neighbor. If this distance (gap) was bigger than one standard deviation of the corresponding parameter, the observations more distant from the mean were excluded from further analyses. 

Using univariate linear regression, we tested for effects of individual factors (mode of delivery, maternal age at delivery, duration of gestation, infants’ gender, birth weight, maternal pre-pregnancy weight, maternal pre-pregnancy body mass index (BMI), maternal CD status, day of lactation, or country of residence) on measured milk analytes. As potentially significant predictors for the multiple regression analysis, we selected the variates that showed Bonferroni corrected *p*-values below 0.2 in both data sets [30]. 

Potentially significant factors were included in the multiple linear regression analysis to test for effects of these factors on the standardized analyte concentrations. Standardization, the transformation of the analytes to have a mean of 0 and standard deviation of 1, was done in order to obtain comparable model estimates. We used weighted effects coding for the categorical variable “country of residence” (each variable is coded such that the estimated effects for each category are to be interpreted as deviations from the weighted mean of the whole data set) to test whether milk components from individual countries differed significantly from the global mean. Subsequently, we utilized analysis of variance (ANOVA) to test for significant differences in the means of the measured analytes across countries. 

For the determination of the relationships among selected analytes, correlation coefficients according to Pearson were calculated for the early and late dataset, respectively.

For the exploration of intra-individual stability of concentrations and percentages, we related data points in the early data set to the corresponding data points in the late data set for the 202 mothers who donated two samples. Intra-individual comparisons were done with paired *t*-tests and correlation coefficients were calculated according to Pearson. 

All statistical analyses were performed with the software R (version 3.0.2., the R foundation for statistical computing). We adjusted the confidence intervals and *p*-values that we report here for multiple testing (41 milk compounds) using Bonferroni’s method. 

## 3. Results

A total of 569 samples from 367 mothers were available. After outlier removal, the early dataset (lactation days 16 to 100) contained results from 319 milk samples with a minimum of 307 values for each analyte. The late dataset (lactation days 101 to 163) with 250 milk samples provided a minimum of 233 values for individual analytes. Early samples were collected on lactation days 42 ± 21 (mean ± SD) and late samples were collected 120 ± 8 days postpartum. A total of 202 of the late samples had an earlier sampled counterpart in the first subset from the same mother. The characteristics of the mothers and their children are summarized in Table 1.

**Figure 1:**
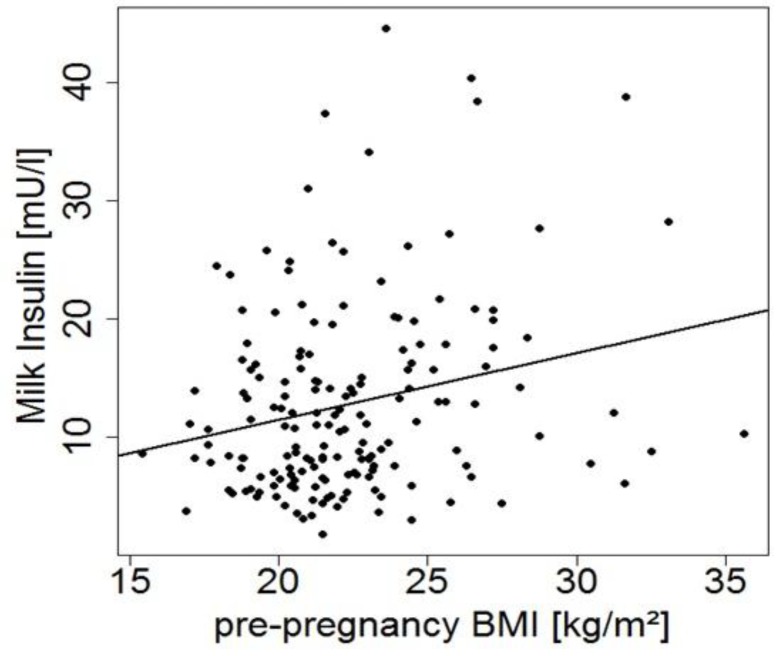
Milk insulin levels in early milk samples versus maternal pre-pregnancy body mass index (BMI) (*r* = 0.24, *p* = 0.002, *n* = 175).

Day of lactation, country of residence, and CD status were identified as potentially relevant variables for milk composition. Pre-pregnancy BMI showed a positive correlation with human milk insulin (Figure 1), but was not considered in other analyses as we have this information only from a small subset of mothers.

### 3.1. Influence of Maternal CD Status, Day of Lactation, and Country of Residence

About half of the participating mothers were CD patients (Table 1). Five out of 184 mothers with CD did not follow a GFD. The early and late dataset showed that the milk of CD negative and positive women was not significantly different regarding the hormone and macronutrient concentrations. Among the FA percentages, only palmitic acid (C16:0) showed significantly decreased percentages in milk of mothers with CD compared with non-CD mothers. Taking all available data into account, palmitic acid contributed 22.3% ± 3.1% to total milk fatty acids in the healthy mothers and 22.0% ± 2.8% in mothers with CD.

**Figure 2:**
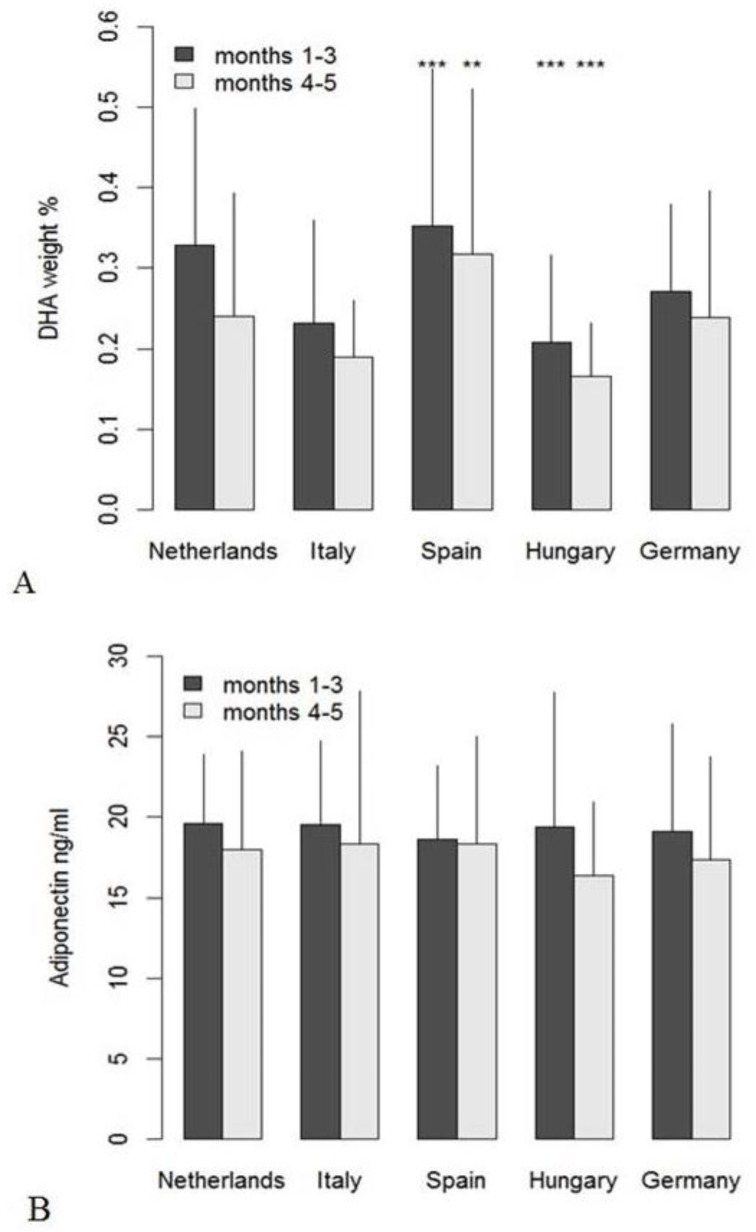
Mean values (+SD) of docosahexaenoic acid (DHA) weight% (**A**) and adiponectin concentration (**B**) per country in early and late milk samples; significant differences from the global means for DHA (months 1–3: 0.29%, months 4–5: 0.24%) and adiponectin (months 1–3: 19.3 ng/mL, months 4–5: 17.6 ng/mL) are indicated as ** for *p* < 0.01 and *** for *p* < 0.001.

Within the first three months of lactation, levels of protein, n-6 LC-PUFA percentages, n-3 eicosatrienoic acid (20:3n-3), capric acid (10:0), lauric acid (12:0), and the monounsaturated fatty acids (MUFA) C20:1n-9 and C24:1n-9 decreased significantly over time (Table 2). Day of lactation did not show significant effects on milk FA composition during months 4 and 5 postpartum (Table 3). During the first three months of lactation, most FA percentages differed significantly across the tested countries (Table 4, docosahexaenoic acid (DHA) in Figure 2A), and long-chain FA also differed by country in late samples (Table 5, DHA in Figure 2A). Comparisons of the individual FA between countries identified a huge number of differences, which were mostly similar in the early and the late data set (Table 4 and Table 5). In the case of DHA, the mean value found in the early samples from Hungary was significantly lower than in the sample from all other countries, and in the late samples, values for Italy and Hungary were similarly low. This is also reflected in 57% and 73%, respectively, of Hungarian samples with DHA below 0.2%, while in the whole sample set, only 29% of the early and 51% of the late samples were below 0.2%. The highest DHA percentages were found in the samples from Spain and the Netherlands, where only 15% and 23%, respectively, of the early samples and 37% and 36%, respectively, of the late samples were below 0.2% DHA.

Country effects were less pronounced for hormones (e.g., adiponectin, Figure 2B) and carbohydrates. IGF-II, protein, and total fat concentrations varied by country during the first three months, but not in the later samples (data not shown in detail).

### 3.2. Correlations among Human Milk Components

We focused on correlations that were consistently significant in both the early and late datasets (Table 6). Protein in milk was positively correlated with adiponectin and IGF-II levels. Milk fat content was not significantly related to adiponectin or insulin, but correlated positively with IGF-II and protein. 

### 3.3. Intrainividual Relationships between Early and Late Milk Samples 

**Figure 3:**
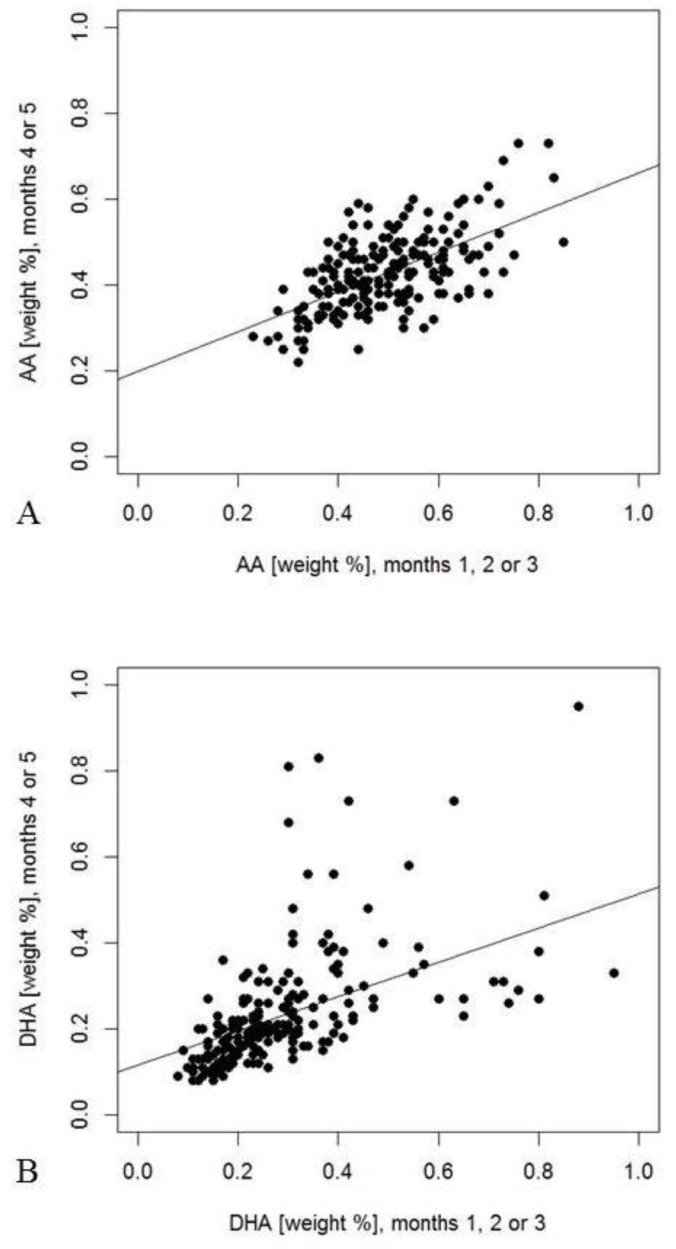
Weight percentages of arachidonic acid (AA) ((**A**), *r* = 0.58, *p* < 0.001) and docosahexaenoic acid (DHA) (**B**), *r* = 0.63, *p* < 0.001) measured in both early and late milk samples. Percentages were calculated for 202 mothers, who had provided samples during the first three months or during the fourth or fifth month of lactation, respectively.

The relationships of the milk components in early and late samples from mothers who donated two samples are summarized in Table 7. The mean difference between the days of collection was 76 days (range 11 to 109 days). Most analytes showed significant correlations (*p* < 0.05, Table 7) with the exception of carbohydrates, protein, as well as caprylic (8:0), arachidic (20:0), and nervonic acid (24:1n9). Adiponectin and IGF-II concentrations showed closer relationships between both time points than insulin. LC-PUFA and odd-chain FA showed the strongest correlations between the two sampling points (e.g., arachidonic acid (AA) and DHA in Figure 3). Significantly lower values in the 4–5 months period than in the early period for protein and most FA agree with the decreases indicated by multiple linear regression analyses during the first three months (Table 7, *t*-test). The exception is capric acid, which decreased during the early period, but was found to be higher in the later period (Table 7). Additionally, DHA showed a significant decrease from the early sample compared with the later sample.

## 4. Discussion

We observed a significant decrease of both milk protein and n-6 LC-PUFA during the first three months of lactation. Variations in the milk FA composition among the different countries were detected. Among the studied compounds, maternal CD only significantly influenced palmitic acid percentage, leading to lower values in the milk of mothers with CD.

In agreement with previous observations [31], milk carbohydrate contents remained stable over time. Human milk protein levels were higher in earlier lactation and continued to decrease beyond month 3 of lactation, similar to previous observations [7,32,33]. IGF-II and adiponectin showed a trend to decrease with time, which concurs with the assumption that IGFs and protein share common determinants or are directly associated [34]. Percentages of most saturated fatty acids SFA and MUFA (except C20:1n-9 and C24:1n-9) did not change with the duration of lactation. In contrast, LC-PUFA levels decreased. Maternal LC-PUFA stores are depleted due to the high requirements during pregnancy [35] and are further consumed during lactation, which leads to delayed recovery of DHA status after birth in breastfeeding women as compared with non-lactating women [36,37]. Diet and endogenous LC-PUFA synthesis from essential FA did not seem to compensate for the high demands. In our study population, milk fat content did not significantly increase with the day of lactation. Therefore, our results do not support the conclusion of Marangoni et al. [38], that lower LC-PUFA percentages with advancing lactation are compensated by an increase of total fat. The discrepant findings could be linked to the heterogeneity in the collection of our milk samples, while Marangoni et al. applied a defined sample collection procedure only allowing hind milk [38]. 

Correlations between early and late samples were low for macronutrients and only significant for milk fat, which may partially be the result of variation in conditions of sample collection. The considerable inter-individual differences of the time span between sample collections may have masked a significant intra individual correlation of protein levels. High PUFA and LC-PUFA correlation coefficients indicate a constancy of dietary habits, which define the individual PUFA and LC-PUFA levels, although levels generally decrease with time. For linoleic acid in human milk, it has been shown that about 30% is directly derived from diet and 70% is contributed by fat storage pools [39]. This also seems to apply to DHA [40] and could explain the high correlation coefficient observed for DHA. Nevertheless, single fish meals can markedly increase DHA in subsequent human milk feeds [3], although day-to-day variation is buffered by the contribution of FA from adipose tissue to milk fat [41,42]. Such single fish meals might have caused some of the observed high DHA-% and this could explain that the correlation between early and late DHA-% was not significant, as only those concentration pairs with at least one value above 0.45% were considered. A corresponding phenomenon was not seen for AA, which is assumed to be mainly contributed by endogenous synthesis from linoleic acid [43].

Our study showed that maternal CD and adherence to a GFD did not have any appreciable effect on milk macronutrients, hormones, and FA, with the exception of a lower palmitic acid percentage in the milk of CD mothers. Endogenous palmitic acid synthesis is stimulated by carbohydrate intake [44]. It is tempting to speculate that avoidance of gluten-containing grain-based foods could lead to a lower contribution of carbohydrates to energy intake, and hence lower palmitic acid synthesis. Comparisons of the diet of CD patients and healthy controls report lower carbohydrate or higher fat intake, respectively, with a GFD than in control groups in some studies, but not in all [45,46,47]. Previous studies have also reported lower n-6 LC-PUFA and higher or lower n-3 LC-PUFA in adult CD patients than in controls [20,21,48]. In this study population from five different countries, we could not find such differences in human milk. Patients with active CD showed higher plasma adiponectin values than healthy controls [49], but this seems not to apply for milk of mothers in remission with CD following a GFD. Olivares et al. had reported lower concentrations of protective immune mediators in milk of women with CD compared with milk from women without CD [22], but we did not identify further nutritionally important effects of CD on human milk. Therefore, breastfeeding can and should be encouraged also in women with CD and a GFD.

Total fat content was only different in the early samples between countries, whereas differences in FA composition were consistently found in both data sets. Potential differences in the lactation day at sample collection were considered in this analysis and do not explain country differences. Total lipid content in human milk is higher in hind-milk than in fore-milk [50]. As fore- or hind-milk collection was not specified in our study, differences of fat content could well be related to differences in sample collection and not reflect different dietary habits. The mode of sample collection does not affect FA composition (weight%), which remains stable during one feeding [51]. The significant variations across countries confirm previous studies that revealed that average human milk FA composition depends on the country and corresponding dietary habits [7,52,53]. While DHA levels were low in milk from Hungarian mothers, Spanish mothers showed the highest DHA levels, which corresponds with previous reports [54,55]. A contribution of at least 0.2% by DHA to total fatty acids in human milk and infant formulas has been considered important for the infant development [56,57], and even 0.3% has been recommended [58]. About 30% of the early samples and 51% of the late samples did not reach the 0.2% level, indicating that sea fish consumption or n-3 LC-PUFA supplement intake should be further encouraged in all included countries, but specifically in Hungary, increased efforts seem required. 

Brenna et al. included 65 studies in their meta-analysis of human milk DHA and AA contents and found, on average, 0.32% ± 0.22% for DHA and 0.47% ± 0.13% for ARA [43]. In our study, DHA values were found to be somewhat lower (early samples 0.29% ± 0.16%, late samples 0.24% ± 015%), but AA values were very close to the reported worldwide average (0.49% ± 0.11%, 0.43% ± 0.09%, respectively). In the whole study population, and stratified according to country, the observed variation was smaller for AA than for DHA, which agrees with the findings in the meta-analysis [43] and the concept that DHA levels in milk depend mainly on dietary intake, while AA is mostly endogenously produced from linoleic acid and levels are related to the desaturase genotype [40]. Although linoleic acid status is usually not found to limit the endogenous AA synthesis [59], the finding that Hungarian samples were highest in linoleic acid and AA could suggest that a very high availability of linoleic acid supports high AA. Comparing α-linolenic and DHA percentages between the countries did not indicate associations between levels, confirming the importance of dietary DHA for appropriate milk levels.

The odd chain tridecanoic and pentadecanoic acids differed between countries and could indicate differences in dairy fat intake, as previously shown for plasma FA [60] or differences in fiber intake [61]. Protein and carbohydrate content did not show consistent differences between countries in the early and late data set. Observational studies in affluent populations and in developing countries have failed to identify dietary factors that influence milk protein or carbohydrates [62,63,64,65].

There was a significant positive correlation between human milk fat and protein. This correlation has been previously described for milk from mothers of very low birth weight infants [66] and in African mothers [67]. A joint regulation of milk protein and milk fat synthesis has been suggested based on in vitro studies [34,68]. The positive correlation of IGF-II with both protein and fat could be of interest, although regulatory effects of IGF-II in the mammary gland were proposed to be much smaller than IGF-I effects [69]. Adiponectin is significantly related to protein, but not to fat content, but models of the actions of adiponectin in the mammary gland have not been developed so far. We found no correlations between insulin and macronutrients in milk. Human milk insulin levels are related to plasma insulin levels and show comparable diurnal variations [13]. Maternal insulin levels were not available, but pre-pregnancy BMI is positively associated with human milk insulin in the present study, comparable to the results of other studies [70,71,72]. 

### Strengths and Limitations

The combined analysis of macronutrients, FA, and selected hormones in our study enabled the parallel examination of day of lactation, country of residence, and CD status, which have not been studied before in large numbers of human milk samples. Some of the results have to be interpreted with caution as spot milk samples without full standardization of sampling have been studied, while 24 h collections of human milk and volume determinations would be more representative, enabling a meaningful consideration of potential effects of mixed feeding. Furthermore, interpretation is limited by partially missing information on maternal anthropometry and the ethnicity of the mothers.

## 5. Conclusions

Milk FA patterns depend on country of residence, which suggests significant dietary influences. In contrast, protein, fat, IGF-II, and adiponectin seem to depend on the individual metabolism. The observed relationships between protein, fat, and IGF-II could agree with an IGF involvement in the regulation of milk synthesis. As no major effects of CD on the studied human milk components were found, breastfeeding should be encouraged in women with CD and a GFD as in the general population.

## Figures and Tables

**Table 1:** Characteristics of participating mothers and their infants.

**Variable**	**M**	**SD**	N *
Age mother, years	33.4	±3.9	357
Gestational age, weeks	39.3	±1.4	366
Pre-pregnancy BMI mother, kg/m²	22.4	±3.4	175
Birth weight, g	3373	±455	364
	**n**	**%**	N *
Mothers with celiac disease	184	50.1	367
Exclusive breastfeeding at 4 months	264	77.9	339
Infant gender female	182	49.6	367

* N corresponds to the number of participants with available information, BMI, body mass index.

**Table 2:** Mean analyte concentrations (±SD) of early samples collected until day 100 of lactation.

	Mean ± SD	CD Mother	Day of Lactation	Country *p*
		β (CI: 0.06%; 99.94%)	β (CI: 0.06%; 99.94%)	
Hormones				
IGF-II, ng/mL	17.41 ± 6.09	−0.048 (−0.423; 0.326)	−0.005 (−0.014; 0.003)	**<0.001**
Insulin, mU/L	12.46 ± 8.19	−0.090 (−0.480; 0.300)	0.003 (−0.006; 0.012)	1
Adiponectin, ng/mL	19.28 ± 6.63	0.134 (−0.267; 0.534)	−0.005 (−0.014; 0.004)	1
macronutrients, g/dL				
Fat	2.2 ± 1.2	−0.202 (−0.600; 0.196)	−0.002 (−0.011; 0.007)	**0.031**
Carbohydrates	6.5 ± 0.4	−0.102 (−0.482; 0.278)	−0.002 (−0.010; 0.007)	0.701
Protein	1.16 ± 0.22	0.081 (−0.276; 0.439)	**−0.015 (−0.023; −0.007)**	**0.049**
Fatty Acids, wt %				
SFA				
C8:0	0.26 ± 0.10	0.129 (−0.258; 0.516)	−0.007 (−0.016; 0.001)	**0.002**
C10:0	1.36 ± 0.32	0.224 (−0.161; 0.609)	**−0.013 (−0.022; −0.004)**	0.122
C12:0	5.37 ± 1.69	0.244 (−0.150; 0.639)	**−0.01 (−0.019; −0.001)**	1
C13:0	0.04 ± 0.01	0.134 (−0.258; 0.526)	−0.002 (−0.011; 0.007)	**0.005**
C14:0	5.76 ± 1.62	0.131 (−0.268; 0.530)	−0.006 (−0.015; 0.003)	**0.031**
C15:0	0.32 ± 0.12	−0.068 (−0.404; 0.269)	0.001 (−0.007; 0.008)	**<0.001**
C16:0	22.16 ± 2.92	**−0.446 (−0.770; −0.122)**	0.002 (−0.005; 0.009)	**<0.001**
C17:0	0.31 ± 0.06	0.020 (−0.342; 0.382)	0.005 (−0.003; 0.013)	**<0.001**
C18:0	7.37 ± 1.33	−0.108 (−0.500; 0.284)	0.007 (−0.002; 0.015)	**<0.001**
C20:0	0.27 ± 0.10	−0.006 (−0.421; 0.409)	0.003 (−0.006; 0.013)	0.821
C22:0	0.10 ± 0.03	0.143 (−0.239; 0.525)	0.004 (−0.004; 0.013)	**<0.001**
C24:0	0.09 ± 0.04	0.181 (−0.184; 0.545)	−0.004 (−0.012; 0.004)	**<0.001**
MUFA				
C14:1	0.23 ± 0.11	−0.102 (−0.442; 0.237)	−0.004 (−0.011; 0.004)	**<0.001**
C15:1	0.07 ± 0.03	0.005 (−0.338; 0.348)	0.001 (−0.007; 0.008)	**<0.001**
C16:1 n-7	2.23 ± 0.69	−0.103 (−0.480; 0.274)	−0.006 (−0.015; 0.002)	**<0.001**
C18:1 n-9	35.33 ± 4.35	0.216 (−0.119; 0.550)	0.005 (−0.003; 0.012)	**<0.001**
C18:1 n-7	1.62 ± 0.25	0.185 (−0.223; 0.593)	−0.007 (−0.016; 0.002)	1
C20:1 n-9	0.46 ± 0.08	0.235 (−0.140; 0.610)	**−0.011 (−0.019; −0.003)**	0.272
C24:1 n-9	0.07 ± 0.02	0.306 (−0.042; 0.653)	**−0.015 (−0.022; −0.007)**	**<0.001**
PUFA				
n6				
C18:2 n-6	13.27 ± 4.16	−0.028 (−0.380; 0.323)	0.002 (−0.005; 0.010)	**<0.001**
C18:3 n-6	0.16 ± 0.05	−0.205 (−0.603; 0.193)	0.000 (−0.009; 0.009)	0.619
C20:2 n-6	0.30 ± 0.09	0.098 (−0.239; 0.435)	**−0.014 (−0.022; −0.007)**	**<0.001**
C20:3 n-6	0.44 ± 0.11	−0.010 (−0.374; 0.354)	**−0.019 (−0.027; −0.011)**	**<0.001**
C20:4 n-6	0.49 ± 0.11	0.066 (−0.294; 0.426)	**−0.013 (−0.021; −0.005)**	**<0.001**
C22:4 n-6	0.11 ± 0.03	0.137 (−0.191; 0.466)	**−0.014 (−0.021; −0.007)**	**<0.001**
C22:5 n-6	0.05 ± 0.02	0.167 (−0.171; 0.506)	**−0.013 (−0.021; −0.006)**	**<0.001**
n3				
C18:3 n-3	0.77 ± 0.39	−0.065 (−0.429; 0.298)	−0.001 (−0.009; 0.007)	**<0.001**
C20:3 n-3	0.05 ± 0.02	0.106 (−0.245; 0.458)	**−0.010 (−0.018; −0.002)**	**<0.001**
C20:5 n-3	0.07 ± 0.05	0.012 (−0.383; 0.408)	−0.005 (−0.014; 0.004)	**<0.001**
C22:5 n-3	0.15 ± 0.05	0.016 (−0.363; 0.396)	−0.007 (−0.016; 0.001)	**<0.001**
C22:6 n-3	0.29 ± 0.16	0.117 (−0.274; 0.508)	−0.009 (−0.017; 0.000)	**<0.001**
n9				
C20:3 n-9	0.02 ± 0.01	0.021 (−0.380; 0.423)	−0.003 (−0.012; 0.006)	**0.018**
Trans FA				
C16:1 trans	0.06 ± 0.02	−0.049 (−0.412; 0.314)	0.005 (−0.003; 0.013)	**<0.001**
C18:1 trans	0.31 ± 0.20	−0.296 (−0.672; 0.080)	0.004 (−0.005; 0.012)	**0.001**
C18:2 trans	0.10 ± 0.04	−0.010 (−0.359; 0.339)	0.002 (−0.005; 0.010)	**<0.001**

Influence of maternal celiac disease (CD) status and day of lactation are indicated by β estimates, and influence of country is indicated by the *p*-values from analysis of variance (ANOVA). Weighted effects coding was used to code the country. *p*-values and 95% confidence intervals were Bonferroni corrected (*n* = 41), resulting in an adjusted 99.88% confidence interval. Significant *p*-values and β estimates are printed in bold. SFA, saturated fatty acids, MUFA, monounsaturated fatty acids; PUFA, polyunsaturated fatty acid; IGF, insulin-like growth factor.

**Table 3:** Mean analyte concentrations (±SD) of late samples collected between days 101 to 163 of lactation.

	Global Mean ± SD	CD Mother	Day of Lactation	Country *p*
		β (CI: 0.06%; 99.94%)	β (CI: 0.06%; 99.94%)	
Hormones				
IGF-II, ng/mL	12.61 ± 3.25	0.055 (−0.393; 0.502)	−0.007 (−0.034; 0.021)	1
Insulin, mU/L	13.67 ± 9.15	−0.207 (−0.600; 0.186)	0.003 (−0.021; 0.027)	1
Adiponectin, ng/mL	17.56 ± 6.26	0.268 (−0.154; 0.689)	−0.008 (−0.034; 0.018)	1
macronutrients, g/dL				
Fat	2.4 ± 1.5	−0.035 (−0.482; 0.413)	0.000 (−0.027; 0.028)	1
Carbohydrates	6.6 ± 0.4	0.123 (−0.258; 0.504)	0.001 (−0.023; 0.024)	1
Protein	0.84 ± 0.18	0.094 (−0.339; 0.527)	−0.014 (−0.041; 0.012)	0.753
Fatty Acids, wt %				
SFA				
C8:0	0.21 ± 0.06	0.142 (−0.297; 0.580)	−0.004 (−0.031; 0.024)	1
C10:0	1.42 ± 0.33	0.317 (−0.119; 0.752)	−0.012 (−0.038; 0.015)	1
C12:0	5.76 ± 1.66	0.181 (−0.261; 0.623)	−0.007 (−0.034; 0.020)	1
C13:0	0.04 ± 0.01	0.031 (−0.386; 0.449)	0.004 (−0.022; 0.030)	**<0.001**
C14:0	6.24 ± 1.71	0.196 (−0.264; 0.656)	0.003 (−0.026; 0.031)	1
C15:0	0.33 ± 0.12	−0.097 (−0.469; 0.276)	0.010 (−0.013; 0.033)	**<0.001**
C16:0	22.33 ± 2.66	**−0.404 (−0.795; −0.012)**	−0.005 (−0.030; 0.019)	**<0.001**
C17:0	0.32 ± 0.06	−0.146 (−0.562; 0.270)	0.005 (−0.021; 0.031)	**<0.001**
C18:0	7.66 ± 1.34	−0.328 (−0.756; 0.100)	−0.002 (−0.029; 0.025)	**<0.001**
C20:0	0.24 ± 0.06	−0.197 (−0.637; 0.243)	−0.005 (−0.032; 0.022)	**0.012**
C22:0	0.09 ± 0.03	0.139 (−0.310; 0.588)	−0.002 (−0.030; 0.026)	**0.004**
C24:0	0.07 ± 0.03	0.212 (−0.240; 0.664)	−0.003 (−0.031; 0.025)	**0.024**
MUFA				
C14:1	0.23 ± 0.11	−0.114 (−0.487; 0.260)	0.005 (−0.018; 0.028)	**<0.001**
C15:1	0.08 ± 0.03	−0.010 (−0.391; 0.370)	0.009 (−0.015; 0.033)	**<0.001**
C16:1 n-7	2.14 ± 0.68	−0.033 (−0.465; 0.398)	0.003 (−0.024; 0.030)	**<0.001**
C18:1 n-9	34.87 ± 4.50	0.271 (−0.114; 0.656)	0.001 (−0.023; 0.025)	**<0.001**
C18:1 n-7	1.54 ± 0.25	0.089 (−0.365; 0.542)	−0.003 (−0.031; 0.025)	1
C20:1 n-9	0.42 ± 0.09	0.259 (−0.188; 0.706)	−0.008 (−0.036; 0.019)	1
C24:1	0.05 ± 0.02	0.304 (−0.147; 0.755)	−0.006 (−0.034; 0.022)	0.223
PUFA				
n6				
C18:2 n-6	12.93 ± 3.72	−0.106 (−0.497; 0.284)	0.005 (−0.019; 0.029)	**<0.001**
C18:3 n-6	0.15 ± 0.04	−0.243 (−0.685; 0.198)	−0.015 (−0.042; 0.012)	0.073
C20:2 n-6	0.25 ± 0.07	−0.068 (−0.456; 0.319)	−0.008 (−0.032; 0.016)	**<0.001**
C20:3 n-6	0.34 ± 0.07	−0.116 (−0.540; 0.308)	−0.025 (−0.052; 0.001)	**<0.001**
C20:4 n-6	0.43 ± 0.09	0.042 (−0.381; 0.466)	−0.016 (−0.043; 0.010)	**<0.001**
C22:4 n-6	0.09 ± 0.03	−0.015 (−0.397; 0.367)	−0.013 (−0.037; 0.011)	**<0.001**
C22:5 n-6	0.04 ± 0.02	0.064 (−0.316; 0.444)	−0.002 (−0.026; 0.022)	**<0.001**
n3				
C18:3 n-3	0.75 ± 0.39	−0.109 (−0.509; 0.291)	0.012 (−0.013; 0.037)	**<0.001**
C20:3 n-3	0.04 ± 0.01	−0.124 (−0.565; 0.317)	0.004 (−0.024; 0.031)	0.111
C20:5 n-3	0.06 ± 0.05	0.089 (−0.368; 0.546)	0.002 (−0.026; 0.031)	**0.027**
C22:5 n-3	0.14 ± 0.05	0.067 (−0.368; 0.502)	−0.001 (−0.028; 0.026)	**<0.001**
C22:6 n-3	0.24 ± 0.15	0.197 (−0.248; 0.641)	0.001 (−0.027; 0.029)	**<0.001**
n9				
C20:3 n-9	0.02 ± 0.01	−0.068 (−0.513; 0.378)	−0.012 (−0.039; 0.016)	**0.018**
Trans FA				
C16:1 trans	0.06 ± 0.02	−0.037 (−0.451; 0.378)	0.013 (−0.013; 0.038)	**<0.001**
C18:1 trans	0.30 ± 0.13	−0.229 (−0.672; 0.215)	−0.002 (−0.029; 0.026)	**0.004**
C18:2 trans	0.11 ± 0.04	−0.044 (−0.435; 0.348)	0.01 (−0.014; 0.035)	**<0.001**

Influence of maternal CD status and day of lactation are indicated by β estimates, and influence of country is indicated by the *p*-values from ANOVA. Weighted effects coding was used to code the country. *p*-values and 95% confidence intervals were Bonferroni corrected (*n* = 41), resulting in an adjusted 99.88% confidence interval. Significant *p*-values and β estimates are printed in bold.

**Table 4:** Human milk fatty acid composition found in the early samples according to the country of residence of the mothers.

	NL	It	ESP	HU	GER
SFA					
C8:0	0.29 ± 0.12 ^ab^	0.22 ± 0.07 ^a^	0.26 ± 0.09 ^c^	0.25 ± 0.12	0.21 ± 0.06 ^bc^
C10:0	1.38 ± 0.35	1.50 ± 0.34	1.37 ± 0.29	1.27 ± 0.33	1.31 ± 0.32
C12:0	5.46 ± 1.84	6.03 ± 1.73	5.23 ± 1.52	5.46 ± 1.96	5.05 ± 1.46
C13:0	0.04 ± 0.01	0.04 ± 0.01	0.04 ± 0.02 ^a^	0.04 ± 0.01 ^b^	0.04 ± 0.01 ^ab^
C14:0	6.06 ± 1.92 ^a^	6.44 ± 1.76 ^b^	5.03 ± 1.42 ^abc^	5.74 ± 1.73	6.33 ± 1.55 ^c^
C15:0	0.31 ± 0.08 ^ab^	0.35 ± 0.09 ^cd^	0.25 ± 0.09 ^a cef^	0.30 ± 0.11 ^eg^	0.45 ± 0.12 ^bdfg^
C16:0	21.54 ± 2.50 ^abc^	24.09 ± 2.37 ^abd^	19.79 ± 2.32 ^def^	22.64 ± 2.50 ^eg^	24.25 ± 2.38 ^cfg^
C17:0	0.29 ± 0.05 ^a^	0.33 ± 0.05	0.30 ± 0.10 ^b^	0.30 ± 0.05 ^c^	0.36 ± 0.06 ^abc^
C18:0	7.38 ± 1.59 ^a^	7.03 ± 1.03 ^b^	6.97 ± 1.15 ^c^	7.40 ± 1.15 ^d^	8.13 ± 1.31 ^abcd^
C20:0	0.30 ± 0.12 ^a^	0.23 ± 0.04 ^a^	0.29 ± 0.13	0.27 ± 0.12	0.27 ± 0.08
C22:0	0.12 ± 0.04 ^abc^	0.08 ± 0.01	0.11 ± 0.08 ^a^	0.09 ± 0.03 ^b^	0.09 ± 0.03 ^c^
C24:0	0.12 ± 0.05 ^abc^	0.07 ± 0.02 ^a^	0.09 ± 0.08	0.08 ± 0.03 ^b^	0.07 ± 0.03 ^c^
MUF A					
C14:1	0.25 ± 0.09 ^abc^	0.24 ± 0.07 ^de^	0.15 ± 0.07 ^adfg^	0.20 ± 0.08 ^bfh^	0.33 ± 0.12 ^cegh^
C15:1	0.07 ± 0.02 ^a^	0.09 ± 0.03 ^bc^	0.06 ± 0.04 ^bd^	0.07 ± 0.03 ^ce^	0.10 ± 0.03 ^ade^
C16:1n-7	2.44 ± 0.78 ^a^	2.20 ± 0.42 ^b^	1.77 ± 0.42 ^abcc^	2.17 ± 0.55 ^ce^	2.58 ± 0.82 ^ce^
C18:1n-9	35.18 ± 3.54 ^ab^	35.58 ± 3.83 ^cd^	38.91 ± 4.33 ^acef^	31.54 ± 3.07 ^bdeg^	34.44 ± 3.35 ^fg^
C18:1n-7	1.64 ± 0.27	1.56 ± 0.19	1.64 ± 0.21	1.59 ± 0.27	1.63 ± 0.28
C20:1n-9	0.47 ± 0.07	0.43 ± 0.06	0.47 ± 0.10	0.43 ± 0.07	0.48 ± 0.12
C24:1	0.08 ± 0.03 ^abc^	0.06 ± 0.02 ^a^	0.07 ± 0.04 ^d^	0.06 ± 0.02 ^b^	0.06 ± 0.02 ^cd^
n-6 PUF A					
C18:2n-6	12.81 ± 3.21 ^abc^	10.46 ± 2.71 ^ade^	13.95 ± 3.94 ^dfg^	16.50 ± 4.27 ^befh^	10.51 ± 2.71 ^cgh^
C18:3n-6	0.15 ± 0.06	0.16 ± 0.05	0.17 ± 0.09	0.17 ± 0.06	0.15 ± 0.04
C20:2n-6	0.29 ± 0.07 ^a^	0.24 ± 0.07 ^bc^	0.33 ± 0.10 ^bd^	0.37 ± 0.08 ^a ce^	0.25 ± 0.06 ^de^
C20:3n-6	0.45 ± 0.09 ^a^	0.44 ± 0.13	0.44 ± 0.12 ^b^	0.47 ± 0.14 ^c^	0.38 ± 0.10 ^abc^
C20:4n-6	0.51 ± 0.12 ^a^	0.45 ± 0.09 ^b^	0.47 ± 0.10 ^c^	0.56 ± 0.12 ^bcd^	0.43 ± 0.09 ^ad^
C22:4n-6	0.11 ± 0.03 ^a^	0.10 ± 0.02 ^b^	0.11 ± 0.08 ^c^	0.14 ± 0.04 ^abcd^	0.09 ± 0.02 ^d^
C22:5n-6	0.05 ± 0.02 ^a^	0.05 ± 0.02	0.05 ± 0.05 ^b^	0.07 ± 0.02 ^abc^	0.04 ± 0.01 ^c^
n-3 PUF A					
C18:3n-3	1.11 ± 0.43 ^abcd^	0.56 ± 0.28 ^ae^	0.60 ± 0.28 ^bf^	0.76 ± 0.34 ^c^	0.80 ± 0.35 ^def^
C20:3n-3	0.06 ± 0.02 ^a^	0.03 ± 0.01 ^a^	0.05 ± 0.06	0.05 ± 0.01	0.05 ± 0.02
C20:5n-3	0.09 ± 0.05 ^ab^	0.05 ± 0.03 ^a^	0.08 ± 0.09 ^c^	0.05 ± 0.04 ^bcd^	0.08 ± 0.05 ^d^
C22:5n-3	0.18 ± 0.05 ^abc^	0.11 ± 0.04 ^ad^	0.14 ± 0.07 ^b^	0.13 ± 0.04 ^ce^	0.16 ± 0.06 ^de^
C22:6n-3	0.31 ± 0.17 ^a^	0.23 ± 0.10 ^b^	0.35 ± 0.20 ^bc^	0.21 ± 0.10 ^a cd^	0.30 ± 0.19 ^d^
n-9 PUF A					
C20:3n-9	0.03 ± 0.01 ^a^	0.03 ± 0.01	0.02 ± 0.01 ^ab^	0.02 ± 0.01	0.03 ± 0.01 ^b^
Tr ^a^ ns F A					
C16:1t	0.06 ± 0.02 ^a^	0.06 ± 0.02	0.06 ± 0.07 ^b^	0.06 ± 0.02 ^c^	0.08 ± 0.03 ^abc^
C18:1t	0.25 ± 0.12 ^a^	0.34 ± 0.36	0.29 ± 0.21 ^b^	0.46 ± 0.43 ^abc^	0.31 ± 0.15 ^c^
C18:2tt	0.10 ± 0.03 ^a^	0.10 ± 0.04 ^b^	0.09 ± 0.10 ^c^	0.09 ± 0.04 ^d^	0.15 ± 0.05 ^abcd^

^a–h^ pairs with common superscripts indicate significant country differences (*p* < 0.05 after Bonferroni adjustment.

**Table 5:** Human milk fatty acid composition found in the late samples according to the country of residence of the mothers.

	NL	It	ESP	HU	GER
SFA					
C8:0	0.21 ± 0.08	0.27 ± 0.17	0.21 ± 0.06	0.22 ± 0.07	0.21 ± 0.06
C10:0	1.34 ± 0.33	1.56 ± 0.34	1.40 ± 0.36	1.40 ± 0.33	1.44 ± 0.38
C12:0	5.46 ± 1.92	6.20 ± 1.77	5.79 ± 1.91	6.00 ± 1.70	5.44 ± 1.54
C13:0	0.04 ± 0.01 ^ab^	0.05 ± 0.02 ^ac^	0.03 ± 0.01 ^cd^	0.04 ± 0.01 ^e^	0.05 ± 0.01 ^bde^
C14:0	6.17 ± 2.23	6.49 ± 1.50	5.77 ± 2.02	5.98 ± 1.78	6.74 ± 1.53
C15:0	0.31 ± 0.08 ^ab^	0.35 ± 0.09 ^cd^	0.25 ± 0.09 ^ace^	0.29 ± 0.11 ^f^	0.45 ± 0.12 ^bdef^
C16:0	21.62 ± 2.39 ^ab^	23.55 ± 2.51 ^c^	19.79 ± 3.58 ^acde^	22.33 ± 2.52 ^df^	24.10 ± 2.19 ^bef^
C17:0	0.31 ± 0.05 ^a^	0.33 ± 0.06 ^b^	0.28 ± 0.07 ^bc^	0.31 ± 0.05 ^d^	0.36 ± 0.06 ^acd^
C18:0	7.63 ± 1.26	7.26 ± 0.97 ^a^	6.87 ± 1.37 ^bc^	7.72 ± 1.25 ^b^	8.35 ± 1.46 ^ac^
C20:0	0.29 ± 0.15 ^ab^	0.33 ± 0.29 ^cde^	0.22 ± 0.06 ^ac^	0.22 ± 0.05 ^bd^	0.24 ± 0.06 ^e^
C22:0	0.11 ± 0.06 ^abc^	0.09 ± 0.02	0.08 ± 0.03 ^a^	0.08 ± 0.03 ^b^	0.09 ± 0.03 ^c^
C24:0	0.09 ± 0.06 ^abc^	0.08 ± 0.03	0.07 ± 0.03 ^a^	0.06 ± 0.02 ^b^	0.06 ± 0.03 ^c^
MUFA					
C14:1	0.24 ± 0.07 ^ab^	0.22 ± 0.08	0.15 ± 0.07 ^ac^	0.20 ± 0.09 ^d^	0.33 ± 0.11 ^bcd^
C15:1	0.07 ± 0.02 ^a^	0.08 ± 0.03 ^b^	0.05 ± 0.02 ^bc^	0.07 ± 0.03 ^d^	0.11 ± 0.03 ^acd^
C16:1n-7	2.25 ± 0.72 ^a^	1.87 ± 0.59 ^b^	1.75 ± 0.51 ^ac^	2.10 ± 0.53 ^d^	2.49 ± 0.75 ^bcd^
C18:1n-9	35.59 ± 3.57 ^ab^	34.92 ± 4.21 ^c^	39.58 ± 9.48 ^acde^	31.31 ± 3.41 ^bdf^	34.07 ± 3.19 ^ef^
C18:1n-7	1.58 ± 0.26	1.45 ± 0.22	1.61 ± 0.45	1.59 ± 0.28	1.50 ± 0.25
C20:1n-9	0.48 ± 0.23	0.38 ± 0.07	0.41 ± 0.10	0.40 ± 0.09	0.44 ± 0.12
C24:1	0.06 ± 0.03	0.06 ± 0.02	0.05 ± 0.02	0.05 ± 0.01	0.05 ± 0.02
n-6 PUFA					
C18:2n-6	12.72 ± 2.66 ^ab^	11.75 ± 3.54 ^c^	12.82 ± 3.51 ^de^	16.42 ± 4.38 ^acdf^	10.35 ± 2.64 ^bef^
C18:3n-6	0.15 ± 0.04	0.16 ± 0.04	0.15 ± 0.04	0.17 ± 0.05	0.14 ± 0.03
C20:2n-6	0.24 ± 0.05 ^ab^	0.24 ± 0.05 ^c^	0.26 ± 0.07 ^de^	0.31 ± 0.07 ^acdf^	0.20 ± 0.05 ^bef^
C20:3n-6	0.33 ± 0.07	0.36 ± 0.05 ^a^	0.34 ± 0.09	0.37 ± 0.10 ^b^	0.30 ± 0.05 ^ab^
C20:4n-6	0.42 ± 0.07 ^a^	0.44 ± 0.08 ^b^	0.40 ± 0.10 ^c^	0.50 ± 0.11 ^abcd^	0.40 ± 0.08 ^d^
C22:4n-6	0.09 ± 0.03 ^a^	0.10 ± 0.02 ^b^	0.08 ± 0.03 ^c^	0.12 ± 0.03 ^abcd^	0.08 ± 0.02 ^d^
C22:5n-6	0.04 ± 0.02 ^a^	0.04 ± 0.01 ^b^	0.04 ± 0.02 ^c^	0.06 ± 0.02 ^abcd^	0.04 ± 0.01 ^d^
n-3 PUFA					
C18:3n-3	1.09 ± 0.43 ^abc^	0.50 ± 0.28 ^ad^	0.55 ± 0.23 ^be^	0.72 ± 0.28 ^c^	0.90 ± 0.48 ^de^
C20:3n-3	0.05 ± 0.02	0.04 ± 0.02	0.04 ± 0.02	0.04 ± 0.01	0.04 ± 0.02
C20:5n-3	0.09 ± 0.11 ^a^	0.05 ± 0.02	0.07 ± 0.05	0.04 ± 0.04 ^ab^	0.08 ± 0.05 ^b^
C22:5n-3	0.18 ± 0.09 ^abc^	0.11 ± 0.03 ^ad^	0.13 ± 0.06 ^b^	0.12 ± 0.03 ^ce^	0.16 ± 0.05 ^de^
C22:6n-3	0.29 ± 0.35	0.19 ± 0.07	0.32 ± 0.20 ^a^	0.18 ± 0.12 ^a^	0.24 ± 0.14
n-9 PUFA					
C20:3n-9	0.02 ± 0.01 ^a^	0.02 ± 0.01	0.02 ± 0.01 ^ab^	0.02 ± 0.01	0.03 ± 0.01 ^b^
Trans FA					
C16:1t	0.06 ± 0.02 ^a^	0.07 ± 0.02	0.05 ± 0.02 ^b^	0.06 ± 0.02 ^c^	0.08 ± 0.02 ^abc^
C18:1t	0.27 ± 0.12 ^a^	0.28 ± 0.11 ^b^	0.26 ± 0.13 ^c^	0.42 ± 0.29 ^abcd^	0.32 ± 0.12 ^d^
C18:2tt	0.12 ± 0.04 ^abc^	0.10 ± 0.04 ^d^	0.08 ± 0.04 ^ae^	0.09 ± 0.03 ^bf^	0.14 ± 0.04 ^cdef^

^a–h^ pairs with common superscripts indicate significant country differences (*p* < 0.05 after Bonferroni adjustment).

**Table 6:** Pearson correlations between the concentrations of macronutrients, hormones, and FA groups (weight%) stratified according to sample collection period.

**Collection during the First Three Months of Lactation (*n* = 319)**								
	IGF-II	Insulin	Adip	Fat	CH	Protein	MUFA	PUFA
Insulin	0.02						
Adip	0.15	−0.05						
Fat	0.36 ***	0.09	0.06					
CH	−0.14	−0.06	−0.12	−0.16				
Protein	0.24 ***	−0.03	0.35 ***	0.23 **	−0.01			
MUFA	−0.12	−0.20 *	−0.1	−0.12	0.12	0.04		
PUFA	0.15	−0.04	0.02	−0.13	0.05	−0.05	−0.33 ***	
SFA	−0.03	0.21 *	0.06	0.21 **	−0.15	0	−0.56 ***	−0.59 ***
**Collection during Months 4 and 5 of Lactation (*n* = 250)**								
	IGF-II	Insulin	Adip	Fat	CH	Protein	MUFA	PUFA
Insulin	0.21 *							
Adip	0.30 ***	0.02						
Fat	0.51 ***	0.17	0.12					
CH	−0.11	0	−0.05	−0.13				
Protein	0.38 ***	0.07	0.30 ***	0.23 *	0.03			
MUFA	−0.1	−0.2	−0.05	−0.09	−0.04	0.02		
PUFA	0.05	−0.07	−0.05	0.12	−0.04	−0.1	−0.34 **	
SFA	0.06	0.25	0.09	0	0.07	0.06	−0.68 ***	−0.46 ***

Note: * *p* < 0.05, ** *p* < 0.01, *** *p* < 0.001 after Bonferroni correction. Significant results are given in bold. Adip = Adiponectin, CH = carbohydrates.

**Table 7:** Comparison of the concentrations measured in the early (from lactation day 16–100) and late samples (from lactation day 101–163) from the mothers who donated two samples (*n* = 202).

	Early (M ± SD)	Late (M ± SD)	*t*-Test *p*	Correlation *r* (*p*)
Hormones and macronutrients				
IGF-II, ng/mL	17.16 ± 5.42	12.62 ± 3.30	**<0.001**	0.303 **(0.001)**
Insulin, mU/L	12.28 ± 8.06	13.72 ± 8.74	0.531	0.480 **(<0.001)**
Adiponectin, ng/mL	19.19 ± 6.06	17.61 ± 6.45	**0.013**	0.466 **(<0.001)**
Fat, g/dL	2.22 ± 1.20	2.54 ± 1.53	0.205	0.346 (<0.001)
Carbohydrates, g/dL	6.54 ± 0.43	6.63 ± 0.38	0.259	0.175 (0.543)
Protein, g/dL	1.15 ± 0.22	0.85 ± 0.18	**<0.001**	0.209 (0.128)
Fatty Acids, wt %				
C8:0	0.25 ± 0.10	0.21 ± 0.06	**<0.001**	0.208 (0.123)
C10:0	1.35 ± 0.32	1.42 ± 0.34	0.305	0.508 **(<0.001)**
C12:0	5.37 ± 1.67	5.74 ± 1.66	**0.032**	0.471 **(<0.001)**
C13:0	0.04 ± 0.01	0.04 ± 0.01	1	0.460 **(<0.001)**
C14:0	5.87 ± 1.66	6.23 ± 1.78	**0.013**	0.596 **(<0.001)**
C15:0	0.34 ± 0.12	0.33 ± 0.12	1	0.676 **(<0.001)**
C16:0	22.34 ± 2.85	22.24 ± 2.72	1	0.591 **(<0.001)**
C17:0	0.32 ± 0.06	0.32 ± 0.06	1	0.504 **(<0.001)**
C18:0	7.35 ± 1.33	7.63 ± 1.35	0.97	0.410 **(<0.001)**
C20:0	0.27 ± 0.09	0.23 ± 0.06	**<0.001**	0.167 (0.995)
C22:0	0.10 ± 0.03	0.09 ± 0.03	**0.002**	0.356 **(<0.001)**
C24:0	0.09 ± 0.03	0.07 ± 0.03	**<0.001**	0.355 **(<0.001)**
C14:1	0.24 ± 0.10	0.23 ± 0.11	1	0.651 **(<0.001)**
C15:1	0.08 ± 0.03	0.08 ± 0.03	1	0.651 **(<0.001)**
C16:1 n-7	2.25 ± 0.69	2.15 ± 0.70	1	0.594 **(<0.001)**
C18:1 n-9	35.25 ± 4.53	35.23 ± 4.54	1	0.605 **(<0.001)**
C18:1 n-7	1.62 ± 0.26	1.54 ± 0.25	**0.004**	0.488 **(<0.001)**
C20:1 n-9	0.46 ± 0.07	0.42 ± 0.09	**<0.001**	0.353 **(<0.001)**
C24:1	0.06 ± 0.02	0.05 ± 0.01	**<0.001**	0.217 (0.129)
C18:2 n-6	13.02 ± 4.19	12.76 ± 3.69	1	0.628 **(<0.001)**
C18:3 n-6	0.16 ± 0.05	0.15 ± 0.04	0.393	0.660 **(<0.001)**
C20:2 n-6	0.30 ± 0.09	0.24 ± 0.06	**<0.001**	0.569 **(<0.001)**
C20:3 n-6	0.44 ± 0.11	0.33 ± 0.07	**<0.001**	0.448 **(<0.001)**
C20:4 n-6	0.49 ± 0.11	0.43 ± 0.08	**<0.001**	0.575 **(<0.001)**
C22:4 n-6	0.11 ± 0.03	0.09 ± 0.03	**<0.001**	0.557 **(<0.001)**
C22:5 n-6	0.05 ± 0.02	0.04 ± 0.02	**<0.001**	0.525 **(<0.001)**
C18:3 n-3	0.77 ± 0.38	0.75 ± 0.40	1	0.514 **(<0.001)**
C20:3 n-3	0.05 ± 0.02	0.04 ± 0.01	**<0.001**	0.388 **(<0.001)**
C20:5 n-3	0.07 ± 0.05	0.06 ± 0.04	1	0.553 **(<0.001)**
C22:5 n-3	0.15 ± 0.05	0.13 ± 0.05	0.48	0.591 **(<0.001)**
C22:6 n-3	0.28 ± 0.16	0.23 ± 0.15	**<0.001**	0.632 **(<0.001)**
C20:3 n-9	0.02 ± 0.01	0.02 ± 0.01	1	0.534 **(<0.001)**
C16:1 trans	0.07 ± 0.03	0.06 ± 0.02	1	0.417 **(<0.001)**
C18:1 trans	0.31 ± 0.20	0.30 ± 0.13	1	0.386 **(<0.001)**
C18:2 trans	0.11 ± 0.04	0.11 ± 0.04	1	0.610 **(<0.001)**

Note: Means between the two samples were compared by paired *t*-test. Correlations between the concentrations of early and late samples were calculated according to Pearson. Significant *p*-values (<0.05 after Bonferroni correction) are given in bold.

## References

[1] Prell C., Koletzko B. (2016). Breastfeeding and Complementary Feeding. Dtsch. Arztebl. Int..

[2] Van Beusekom C., Martini I.A., Rutgers H.M., Boersma E.R., Muskiet F.A. (1990). A carbohydrate-rich diet not only leads to incorporation of medium-chain fatty acids (6:0-14:0) in milk triglycerides but also in each milk-phospholipid subclass. Am. J. Clin. Nutr..

[3] Lauritzen L., Jorgensen M.H., Hansen H.S., Michaelsen K.F. (2002). Fluctuations in human milk long-chain PUFA levels in relation to dietary fish intake. Lipids.

[4] Grote V., Verduci E., Scaglioni S., Vecchi F., Contarini G., Giovannini M., Koletzko B., Agostoni C. (2015). Breast milk composition and infant nutrient intakes during the first 12 months of life. Eur. J. Clin. Nutr..

[5] Armand M., Bernard J.Y., Forhan A., Heude B., Charles M.A., EDEN Mother-Child Cohort Study Group (2018). Maternal nutritional determinants of colostrum fatty acids in the EDEN mother-child cohort. Clin. Nutr..

[6] Gidrewicz D.A., Fenton T.R. (2014). A systematic review and meta-analysis of the nutrient content of preterm and term breast milk. BMC Pediatrics.

[7] Jensen R.G. (1995). Handbook of Milk Composition.

[8] Bravi F., Wiens F., Decarli A., Dal Pont A., Agostoni C., Ferraroni M. (2016). Impact of maternal nutrition oh breast-milk composition: A systematic review. Am. J. Clin. Nutr..

[9] Nasser R., Stephen A.M., Goh Y.K., Clandinin M.T. (2010). The effect of a controlled manipulation of maternal dietary fat intake on medium and long chain fatty acids in human breast milk in Saskatoon, Canada. Int. Breastfeed. J..

[10] Ballard O., Morrow A.L. (2013). Human Milk Composition. Pediatric Clin. N. Am..

[11] Grosvenor C.E., Picciano M.F., Baumrucker C.R. (1993). Hormones and growth factors in milk. Endocr. Rev..

[12] Newburg D.S., Woo J.G., Morrow A.L. (2010). Characteristics and Potential Functions of Human Milk Adiponectin. J. Pediatr..

[13] Cevreska S., Kovacev V.P., Stankovski M., Kalamaras E. (1975). The presence of immunologically reactive insulin in milk of women, during the first week of lactation and its relation to changes in plasma insulin concentration. God. Zb. Med. Fak. Skopje.

[14] Woo J.G., Guerrero M.L., Altaye M., Ruiz-Palacios G.M., Martin L.J., Dubert-Ferrandon A., Newburg D.S., Morrow A.L. (2009). Human milk adiponectin is associated with infant growth in two independent cohorts. Breastfeed. Med..

[15] Prentice P., Ong K.K., Schoemaker M.H., van Tol E.A., Vervoort J., Hughes I.A., Acerini C.L., Dunger D.B. (2016). Breast milk nutrient content and infancy growth. Acta Paediatr..

[16] Heinig M.J., Nommsen L.A., Peerson J.M., Lonnerdal B., Dewey K.G. (1993). Energy and protein intakes of breast-fed and formula-fed infants during the first year of life and their associationwith growth velocity: The DARLING Study. Am. J. Clin. Nutr..

[17] Eriksen K.G., Christensen S.H., Lind M.V., Michaelsen K.F. (2018). Human milk composition and infant growth. Curr. Opin. Clin. Nutr. Metab. Care.

[18] Green P.H.R., Cellier C. (2007). Medical progress: Celiac disease. N. Engl. J. Med..

[19] Thompson T., Dennis M., Higgins L.A., Lee A.R., Sharrett M.K. (2005). Gluten-free diet survey: Are Americans with coeliac disease consuming recommended amounts of fibre, iron, calcium and grain foods?. J. Hum. Nutr. Diet..

[20] Van Hees N.J.M., Giltay E.J., Geleijnse J.M., Janssen N., van der Does W. (2014). DHA Serum Levels Were Significantly Higher in Celiac Disease Patients Compared to Healthy Controls and Were Unrelated to Depression. PLoS ONE.

[21] Russo F., Chimienti G., Clemente C., Ferreri C., Orlando A., Riezzo G. (2017). A possible role for ghrelin, leptin, brain-derived neurotrophic factor and docosahexaenoic acid in reducing the quality of life of coeliac disease patients following a gluten-free diet. Eur. J. Nutr..

[22] Olivares M., Albrecht S., De Palma G., Ferrer M.D., Castillejo G., Schols H.A., Sanz Y. (2015). Human milk composition differs in healthy mothers and mothers with celiac disease. Eur. J. Nutr..

[23] Hogen Esch C.E., Rosen A., Auricchio R., Romanos J., Chmielewska A., Putter H., Ivarsson A., Szajewska H., Koning F., Wijmenga C. (2010). The PreventCD Study design: towards new strategies for the prevention of coeliac disease. Eur. J. Gastroenterol. Hepatol..

[24] Vriezinga S.L., Auricchio R., Bravi E., Castillejo G., Chmielewska A., Crespo Escobar P., Kolacek S., Koletzko S., Korponay-Szabo I.R., Mummert E. (2014). Randomized feeding intervention in infants at high risk for celiac disease. N. Engl. J. Med..

[25] Hellmuth C., Uhl O., Demmelmair H., Grunewald M., Auricchio R., Castillejo G., Korponay-Szabo I.R., Polanco I., Roca M., Vriezinga S.L. (2018). The impact of human breast milk components on the infant metabolism. PLoS ONE.

[26] Casadio Y.S., Williams T.M., Lai C.T., Olsson S.E., Hepworth A.R., Hartmann P.E. (2010). Evaluation of a mid-infrared analyzer for the determination of the macronutrient composition of human milk. J. Hum. Lact..

[27] Polberger S., Lönnerdal B. (1993). Simple and Rapid Macronutrient Analysis of Human Milk for Individualized Fortification: Basis for Improved Nutritional Management of Very-Low-Birth-Weight Infants?. J. Pediatr. Gastr. Nutr..

[28] Stimming M., Mesch C.M., Kersting M., Kalhoff H., Demmelmair H., Koletzko B., Schmidt A., Bohm V., Libuda L. (2014). Vitamin E content and estimated need in German infant and follow-on formulas with and without long-chain polyunsaturated fatty acids (LC-PUFA) enrichment. J. Agric. Food Chem..

[29] Goelz R., Hihn E., Hamprecht K., Dietz K., Jahn G., Poets C., ElmLinger M. (2009). Effects of Different CMV-Heat-Inactivation-Methods on Growth Factors in Human Breast Milk. Pediatr. Res..

[30] Vittinghoff E., Glidden D., Shiboski S., McCulloch C. (2005). Regression Methods in Biostatistics: Linear, Logistic, Survival, and Repeated Measures Models.

[31] Mitoulas L.R., Kent J.C., Cox D.B., Owens R.A., Sherriff J.L., Hartmann P.E. (2002). Variation in fat, lactose and protein in human milk over 24h and throughout the first year of lactation. Br. J. Nutr..

[32] Shehadeh N., Aslih N., Shihab S., Werman M.J., Sheinman R., Shamir R. (2006). Human milk beyond one year post-partum: Lower content of protein, calcium, and saturated very long-chain fatty acids. J. Pediatrics.

[33] Lonnerdal B., Erdmann P., Thakkar S.K., Sauser J., Destaillats F. (2017). Longitudinal evolution of true protein, amino acids and bioactive proteins in breast milk: A developmental perspective. J. Nutr. Biochem..

[34] Anderson S.M., Rudolph M.C., McManaman J.L., Neville M.C. (2007). Secretory activation in the mammary gland: It’s not just about milk protein synthesis. Breast Cancer Res..

[35] Herrera E., Amusquivar E., López-Soldado I., Ortega H. (2006). Maternal lipid metabolism and placental lipid transfer. Horm. Res. Paediatr..

[36] Hornstra G. (2000). Essential fatty acids in mothers and their neonates. Am. J. Clin. Nutr..

[37] Jorgensen M.H., Nielsen P.K., Michaelsen K.F., Lund P., Lauritzen L. (2006). The composition of polyunsaturated fatty acids in erythrocytes of lactating mothers and their infants. Matern. Child Nutr..

[38] Marangoni F., Agostoni C., Lammardo A.M., Giovannini M., Galli C., Riva E. (2000). Polyunsaturated fatty acid concentrations in human hindmilk are stable throughout 12-months of lactation and provide a sustained intake to the infant during exclusive breastfeeding: An Italian study. Br. J. Nutr..

[39] Demmelmair H., Baumheuer M., Koletzko B., Dokoupil K., Kratl G. (1998). Metabolism of U13C-labeled linoleic acid in lactating women. J. Lipid Res..

[40] Demmelmair H., Koletzko B. (2018). Lipids in human milk. Best Pract. Res. Clin. Endocrinol. Metab..

[41] Innis S.M. (2007). Fatty acids and early human development. Early Hum. Dev..

[42] Demmelmair H., Sauerwald T., Fidler N., Baumheuer M., Koletzko B. (2001). Polyunsaturated fatty acid metabolism during lactation. World Rev. Nutr. Diet..

[43] Brenna J.T., Varamini B., Jensen R.G., Diersen-Schade D.A., Boettcher J.A., Arterburn L.M. (2007). Docosahexaenoic and arachidonic acid concentrations in human breast milk worldwide. Am. J. Clin. Nutr..

[44] Hudgins L.C., Hellerstein M., Seidman C., Neese R., Diakun J., Hirsch J. (1996). Human fatty acid synthesis is stimulated by a eucaloric low fat, high carbohydrate diet. J. Clin. Investig..

[45] Kinsey L., Burden S.T., Bannerman E. (2008). A dietary survey to determine if patients with coeliac disease are meeting current healthy eating guidelines and how their diet compares to that of the British general population. Eur. J. Clin. Nutr..

[46] Melini V., Melini F. (2019). Gluten-Free Diet: Gaps and Needs for a Healthier Diet. Nutrients.

[47] Capristo E., Addolorato G., Mingrone G., De Gaetano A., Greco A.V., Tataranni P.A., Gasbarrini G. (2000). Changes in body composition, substrate oxidation, and resting metabolic rate in adult celiac disease patients after a 1-y gluten-free diet treatment. Am. J. Clin. Nutr..

[48] Solakivi T., Kaukinen K., Kunnas T., Lehtimaki T., Maki M., Nikkari S.T. (2009). Serum fatty acid profile in celiac disease patients before and after a gluten-free diet. Scand. J. Gastroenterol..

[49] Russo F., Chimienti G., Clemente C., D’Attoma B., Linsalata M., Orlando A., De Carne M., Cariola F., Semeraro F.P., Pepe G. (2013). Adipokine profile in celiac patients: Differences in comparison with patients suffering from diarrhea-predominant IBS and healthy subjects. Scand. J. Gastroenterol..

[50] Mizuno K., Nishida Y., Taki M., Murase M., Mukai Y., Itabashi K., Debari K., Iiyama A. (2009). Is increased fat content of hindmilk due to the size or the number of milk fat globules?. Int. Breastfeed. J..

[51] Emery W.B., Canolty N.L., Aitchison J.M., Dunkley W.L. (1978). Influence of sampling on fatty acid composition of human milk. Am. J. Clin. Nutr..

[52] Yuhas R., Pramuk K., Lien E.L. (2006). Human milk fatty acid composition from nine countries varies most in DHA. Lipids.

[53] Keikha M., Bahreynian M., Saleki M., Kelishadi R. (2017). Macro- and Micronutrients of Human Milk Composition: Are They Related to Maternal Diet? A Comprehensive Systematic Review. Breastfeed. Med..

[54] Barreiro R., Diaz-Bao M., Cepeda A., Regal P., Fente C.A. (2018). Fatty acid composition of breast milk in Galicia (NW Spain): A cross-country comparison. Prostaglandins Leukot. Essent. Fatty Acids.

[55] Decsi T., Olah S., Molnar S., Burus I. (2000). Low contribution of docosahexaenoic acid to the fatty acid composition of mature human milk in Hungary. Adv. Exp. Med. Biol..

[56] Koletzko B., Lien E., Agostoni C., Bohles H., Campoy C., Cetin I., Decsi T., Dudenhausen J.W., Dupont C., Forsyth S. (2008). The roles of long-chain polyunsaturated fatty acids in pregnancy, lactation and infancy: Review of current knowledge and consensus recommendations. J. Perinat. Med..

[57] FAO (2010). Fats and Fatty Acids in Human Nutrition—Report of an Expert Consultation.

[58] Koletzko B., Boey C.C., Campoy C., Carlson S.E., Chang N., Guillermo-Tuazon M.A., Joshi S., Prell C., Quak S.H., Sjarif D.R. (2014). Current information and Asian perspectives on long-chain polyunsaturated fatty acids in pregnancy, lactation, and infancy: Systematic review and practice recommendations from an early nutrition academy workshop. Ann. Nutr. Metab..

[59] Demmelmair H., MacDonald A., Kotzaeridou U., Burgard P., Gonzalez-Lamuno D., Verduci E., Ersoy M., Gokcay G., Alyanak B., Reischl E. (2018). Determinants of Plasma Docosahexaenoic Acid Levels and Their Relationship to Neurological and Cognitive Functions in PKU Patients: A Double Blind Randomized Supplementation Study. Nutrients.

[60] Brevik A., Veierod M.B., Drevon C.A., Andersen L.F. (2005). Evaluation of the odd fatty acids 15:0 and 17:0 in serum and adipose tissue as markers of intake of milk and dairy fat. Eur. J. Clin. Nutr..

[61] Weitkunat K., Schumann S., Nickel D., Hornemann S., Petzke K.J., Schulze M.B., Pfeiffer A.F.H., Klaus S. (2017). Odd-chain fatty acids as a biomarker for dietary fiber intake: A novel pathway for endogenous production from propionate. Am. J. Clin. Nutr..

[62] Nommsen L.A., Lovelady C.A., Heinig M.J., Lonnerdal B., Dewey K.G. (1991). Determinants of energy, protein, lipid, and lactose concentrations in human milk during the first 12 mo of lactation: The DARLING Study. Am. J. Clin. Nutr..

[63] Yang T., Zhang Y., Ning Y., You L., Ma D., Zheng Y., Yang X., Li W., Wang J., Wang P. (2014). Breast milk macronutrient composition and the associated factors in urban Chinese mothers. Chin. Med. J..

[64] Quinn E.A., Largado F., Power M., Kuzawa C.W. (2012). Predictors of breast milk macronutrient composition in Filipino mothers. Am. J. Hum. Biol..

[65] Bzikowska-Jura A., Czerwonogrodzka-Senczyna A., Oledzka G., Szostak-Wegierek D., Weker H., Wesolowska A. (2018). Maternal Nutrition and Body Composition During Breastfeeding: Association with Human Milk Composition. Nutrients.

[66] Weber A., Loui A., Jochum F., Buhrer C., Obladen M. (2001). Breast milk from mothers of very low birthweight infants: Variability in fat and protein content. Acta Paediatr..

[67] Roels O.A. (1958). Correlation between the fat and the protein content of human milk. Nature.

[68] Qi L., Yan S., Sheng R., Zhao Y., Guo X. (2014). Effects of Saturated Long-chain Fatty Acid on mRNA Expression of Genes Associated with Milk Fat and Protein Biosynthesis in Bovine Mammary Epithelial Cells. Asian Australas. J. Anim. Sci..

[69] Prosser C.G. (1996). Insulin-like growth factors in milk and mammary gland. J. Mammary Gland Biol. Neoplasia.

[70] Ahuja S., Boylan M., Hart S.L., Román-Shriver C., Spallholz J.E., Pence B.C., Sawyer B.G. (2011). Glucose and Insulin Levels are Increased in Obese and Overweight Mothers’ Breast-Milk. Food Nutr. Sci..

[71] Ley S.H., Hanley A.J., Sermer M., Zinman B., O’Connor D.L. (2012). Associations of prenatal metabolic abnormalities with insulin and adiponectin concentrations in human milk. Am. J. Clin. Nutr..

[72] Demmelmair H., Koletzko B. (2017). Variation of Metabolite and Hormone Contents in Human Milk. Clin. Perinatol..

